# Optimising plasma levels of clozapine during metabolic interactions: a review and case report with adjunct rifampicin treatment

**DOI:** 10.1186/s12888-015-0536-4

**Published:** 2015-08-12

**Authors:** Siobhan Gee, Thomas Dixon, Mary Docherty, Sukhwinder S Shergill

**Affiliations:** Bethlem Royal Hospital, South London and Maudsley NHS Foundation Trust, Monks Orchard Road, Beckenham, BR3 3BX UK; Springfield University Hospital, South West London and St George’s Mental Health NHS Trust, 61 Glenburnie Road, London, SW17 7DJ UK; Institute of Psychiatry, Psychology and Neuroscience, King’s College London, 16 De Crespigny Park, London, SE5 8AF UK

**Keywords:** Clozapine, Rifampicin, Schizophrenia, Schizoaffective disorder, Tuberculosis

## Abstract

**Background:**

Clozapine is the only licensed medication for treatment-resistant schizophrenia. The metabolism of clozapine is affected by multiple pharmacokinetic interactions, so the co-administration of adjunct medications can have a significant clinical effect. The anti- tuberculosis medication rifampicin is a potent inducer of the cytochrome P450 system and therefore can cause a reduction in the plasma concentration of clozapine. There is limited clinical evidence regarding co-administration of these medications; in particular there is a lack of data regarding the effect on plasma clozapine levels, which is the key factor determining clinical efficacy. This is clinically relevant given evidence of an increased risk of tuberculosis in patients with schizophrenia.

**Case Presentation:**

We present a case of a 28 year old British man with a diagnosis of schizoaffective disorder who presented with persistent psychotic symptoms. He developed a systemic inflammatory condition, diagnosed as tuberculosis, and was commenced on a six month course of treatment that included rifampicin. This case presents comprehensive data to illustrate the effect on clozapine plasma levels of a complete course of tuberculosis therapy.

**Conclusion:**

This case report provides guidance to clinicians in managing drug interactions between clozapine and rifampicin to enable safe and effective treatment. The co-administration of these medications is likely to increase as the existing underuse of clozapine is recognised whilst the incidence of tuberculosis increases.

## Background

Clozapine is the only drug treatment licensed for use in treatment-resistant schizophrenia [1]. The UK National Institute for Health and Care Excellence recommends that clozapine should be offered to people with schizophrenia whose illness has not sufficiently responded to treatment, despite the sequential use of adequate doses of two different antipsychotic drugs [2], at least one of which should be a non-clozapine second-generation antipsychotic [3].

Despite clear guidance in the UK, there is widespread evidence that clozapine is underused [4, 5]. While relatively few first onset patients with schizophrenia fail to show a positive response to their first two antipsychotics [6], later in the illness up to 30 % of patients fail to show an adequate response, of whom 30–60 % would respond to clozapine if offered [7]. The main reasons for not prescribing clozapine may include clinician and patient concern about the essential regular blood tests and potential side effects [8]. Approximately 0.8 % of patients who take clozapine will develop agranulocytosis [3, 9]. Mandatory blood monitoring protocols provide timely identification of any significant haematological abnormalities. The most common adverse effects of clozapine are increased sedation, hypersalivation, constipation, weight gain, tachycardia, hypotension and seizures, but there are clear management strategies for these [10]. Unlike the risk of agranulocytosis, some of these side effects, such as sedation and seizures, are known to be plasma-level dependent.

The key variable determining the ultimate efficacy of clozapine and the severity of some adverse effects is the plasma concentration, which is determined by two main factors: the dose of clozapine taken by the patient, and the speed of its metabolism. There is a high inter-patient variability in the relationship between clozapine dose and serum level, reflecting variability in its metabolism between patients [11, 12]. Measurement of serum levels is therefore important to ensure both an optimal therapeutic level and to minimise the risk of toxic side effects. Most studies of patients with treatment-resistant schizophrenia indicate that the threshold for response is in the range 0.35–0.42 mg/L [13], although others suggest a higher upper limit of 0.50 mg/L [14]. Tolerability can be improved by employing a gradual initial titration regimen, with subsequent dose changes made on the basis of adverse side effects, effectiveness and plasma levels.

**Fig. 1:**
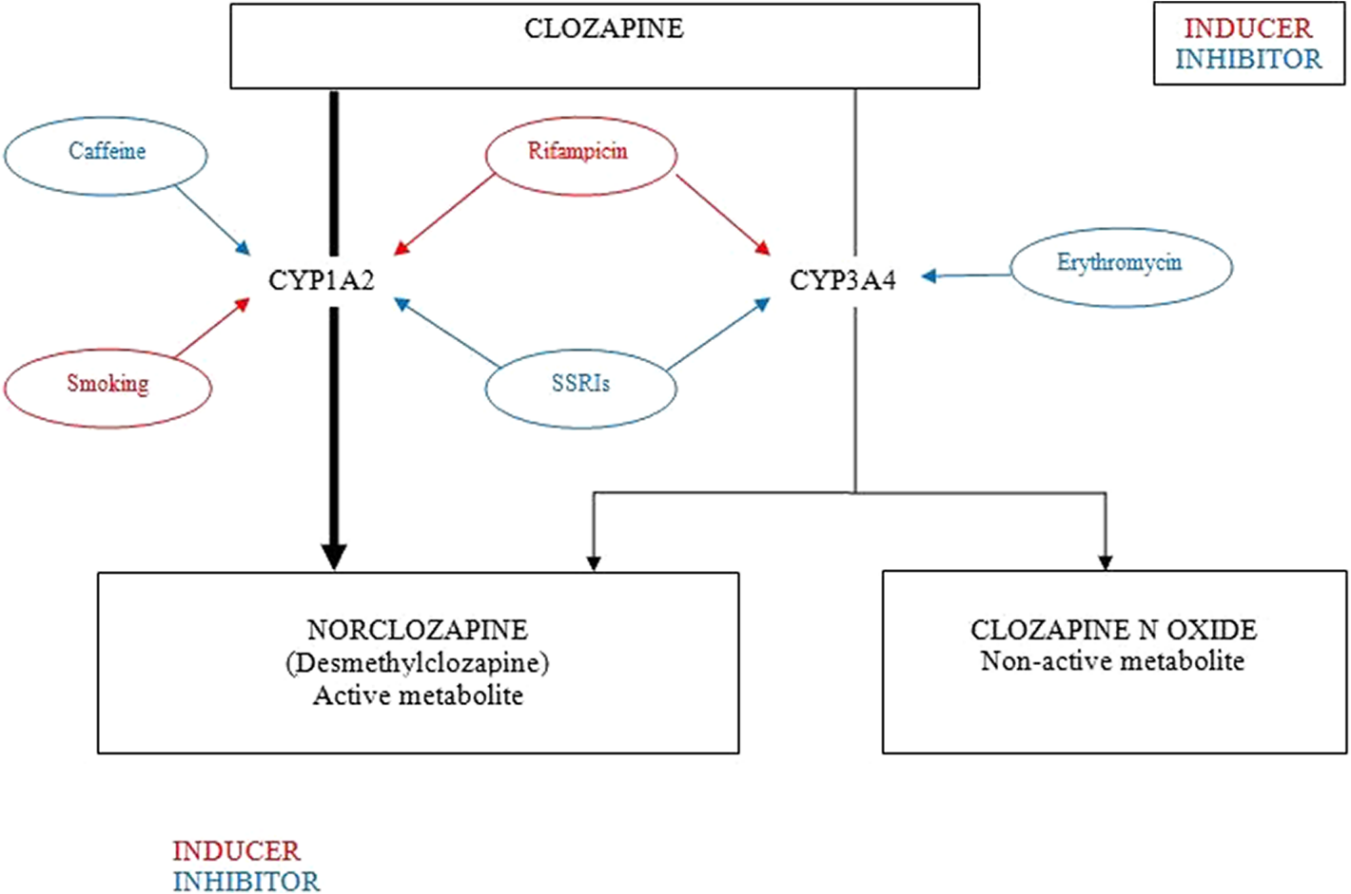
The main clozapine metabolic pathways and pharmacokinetic interactions

Clozapine undergoes complex hepatic metabolism involving multiple cytochrome P450 (CYP) isoforms, the enzyme variants that catalyse biotransformation reactions (Fig. 1). It is primarily metabolised by the CYP1A2 isoform, but CYP3A4 and to a lesser extent CYP2C9, CYP2C19 and CYP2D6 are also involved [15]. Consequently, clozapine is involved in significant pharmacokinetic interactions when co-administered with other drugs. CYP1A2 isoforms are inhibited by some antidepressants (including fluoxetine and fluvoxamine) and caffeine, resulting in increased clozapine plasma concentrations [16]. Conversely, CYP1A2 isoforms may be induced by some antibiotics (erythyromycin) and cigarette smoke (through the effect of aromatic hydrocarbons), which will decrease clozapine plasma concentrations [17, 18]. The antimycobacterial agent rifampicin is an inducer of CYP1A2 and CYP3A4 [15, 19]; adjunct treatment with rifampicin would be expected to decrease serum concentrations of clozapine. The consistency of CYP-mediated drug interactions with clozapine is not always easy to predict [20].

Whilst tuberculosis (TB) has been considered to be largely restricted to lower income parts of the world, increased global travelling has caused a resurgence of this illness in higher income countries, particularly in certain urban centres. The incidence of TB is higher in people with schizophrenia than in the general population [21], and there are common risk factors for the development of severe mental illness and the development of multi-drug resistant TB (homelessness, substance abuse and imprisonment) [22].

First-line medication for TB is a quadruple therapy regimen of rifampicin, isoniazid, pyrazinamide and ethambutol for two months, followed by a four month continuation phase of rifampicin and isoniazid [1]. The literature on the interaction between rifampicin and clozapine is limited to three case reports; the first reports a halving of haloperidol plasma concentrations, and increased clearance of diazepam by 300 % and nitrazepam by 83 %, upon commencing rifampicin [23]. The second describes a patient stabilised on clozapine who experienced a significant decrease in his clozapine plasma concentration when rifampicin was added to therapy, with an exacerbation of psychotic symptoms [24]. The decrease in clozapine concentration was seen 2–3 weeks after the initiation of rifampicin and levels rose 3 days after rifampicin treatment was discontinued, although the concomitant substitution with the enzyme inhibitor ciprofloxacin may also have contributed to this effect. A third report describes a notable deterioration in psychotic symptoms in a patient with schizophrenia during the six months of rifampicin therapy [25]; no data on clozapine levels were available, but the deterioration in symptoms persisted despite an increase in the dose of clozapine from 300 mg to 550 mg/day.

Whilst literature describing the interaction between clozapine and rifampicin is limited to the three case reports described above, many more reports demonstrate the effect of adding rifampicin to other psychotropics. Given than rifampicin (as stated above) induces CYP1A2 and 3A4, a reduction in plasma concentrations of medications that are metabolised by these enzymes should be expected. As well as clozapine, the antipsychotic olanzapine is also a substrate for CYP1A2 (and, like clozapine, this isoform is the predominant metabolic pathway for the drug) [2]. Interestingly, reports of reduction in olanzapine plasma concentrations due to co-administration of rifampicin are absent from the literature – perhaps because measurement of olanzapine levels is not usually considered part of routine clinical practice.

Many different psychotropics are metabolised by CYP3A4, including aripiprazole, methadone, mirtazapine, clonazepam, quetiapine and zopiclone. Studies in healthy subjects clearly establish the pharmacokinetic interaction between rifampicin and many of the benzodiazepines and non-benzodiazepine hypnotics [26–28], the antidepressant bupropion [29] and the anxiolytic buspirone [30]. Case reports describe reductions in plasma concentrations of psychotropics when combined with rifampicin even where the rifampicin inhibited CYP enzyme might not be considered as the main metabolic pathway for the drug – this is the case for amitriptyline [31], sertraline [32], citalopram [33] and haloperidol [34]. Interactions with other psychotropics, including quetiapine and aripiprazole, are not described in the literature but are limited to supposition based on the metabolism of the drug.

Interactions between rifampicin and psychotropics are not limited to mechanisms involving CYP enzymes. A study in healthy subjects [35] and a case report [36] describe reductions in lamotrigine plasma concentrations caused by rifampicin, possibly due to increased lamotrigine glucuronidation via glucuronyltransferases. CYP3A4 is a minor metabolic pathway for the antipsychotic risperidone, but two studies describe marked decreases in plasma concentrations when rifampicin is co-administered [37, 38]. The authors suggest the possible role of P-glycoprotein in this interaction.

There is little guidance available to aid clinicians faced with patients with schizophrenia on clozapine treatment, who are also initiating concurrent treatment for TB. This is an increasing issue for deprived urban areas in cities with large migrant populations, and for those patients who are immunocompromised or abusing illicit drugs [22, 39]. Here we discuss the changes in the plasma concentration of clozapine using an illustrative case of a patient stabilised on clozapine who commenced a course of TB treatment which included rifampicin.

## Case Presentation

The National Psychosis Service at the Maudsley Hospital (South London and Maudsley NHS Foundation Trust, (SLaM)) is a tertiary referral centre for patients with complex treatment- resistant psychotic illnesses, often with significant co-morbid physical health conditions [40]. Clozapine is therefore frequently prescribed in combination with a range of other physical health medications. The recommended therapeutic range for clozapine plasma levels at SLaM is 0.35 to 0.50 mg/L although occasionally higher plasma levels may be targeted with adequate seizure prophylaxis.

Mr X is a 28 year old British man of African heritage who has been known to mental health services since the age of 18, with a diagnosis of schizoaffective disorder. He had spent over 4 years as an inpatient in hospital, spread over 9 admissions, and had been an inpatient for two years before being transferred to the National Psychosis Service. He presented with manic and depressive phases to his illness, both of which had been associated with auditory and visual hallucinations, bizarre delusional beliefs and catatonia.

During the course of his illness he has been treated with both first and second generation antipsychotics for over 6 weeks duration at maximum recommended doses with a limited response. He had previously shown some positive response to clozapine during an earlier period of illness, but this was stopped due to concerns about developing diabetes. He was recommenced on clozapine treatment soon after admission to the National Psychosis Unit. His other psychotropic medication included fluoxetine for depressive symptoms and lamotrigine as both a mood stabiliser and for seizure prophylaxis.

Over the last two years he had been experiencing a chronic systemic inflammatory condition. He was initially transferred to a general hospital after developing signs of abdominal sepsis and blood tests that revealed microcytic anaemia and a significant inflammatory response. Despite an extensive range of investigations, including blood and sputum samples and an inconclusive lymph node biopsy a definitive cause for his presentation was not identified. Various specialists concluded that he should be treated empirically for abdominal TB. A six month course of rifampicin and isoniazid was commenced together with pyrazinamide and moxifloxacin for the first two of those months.

**Fig. 2:**
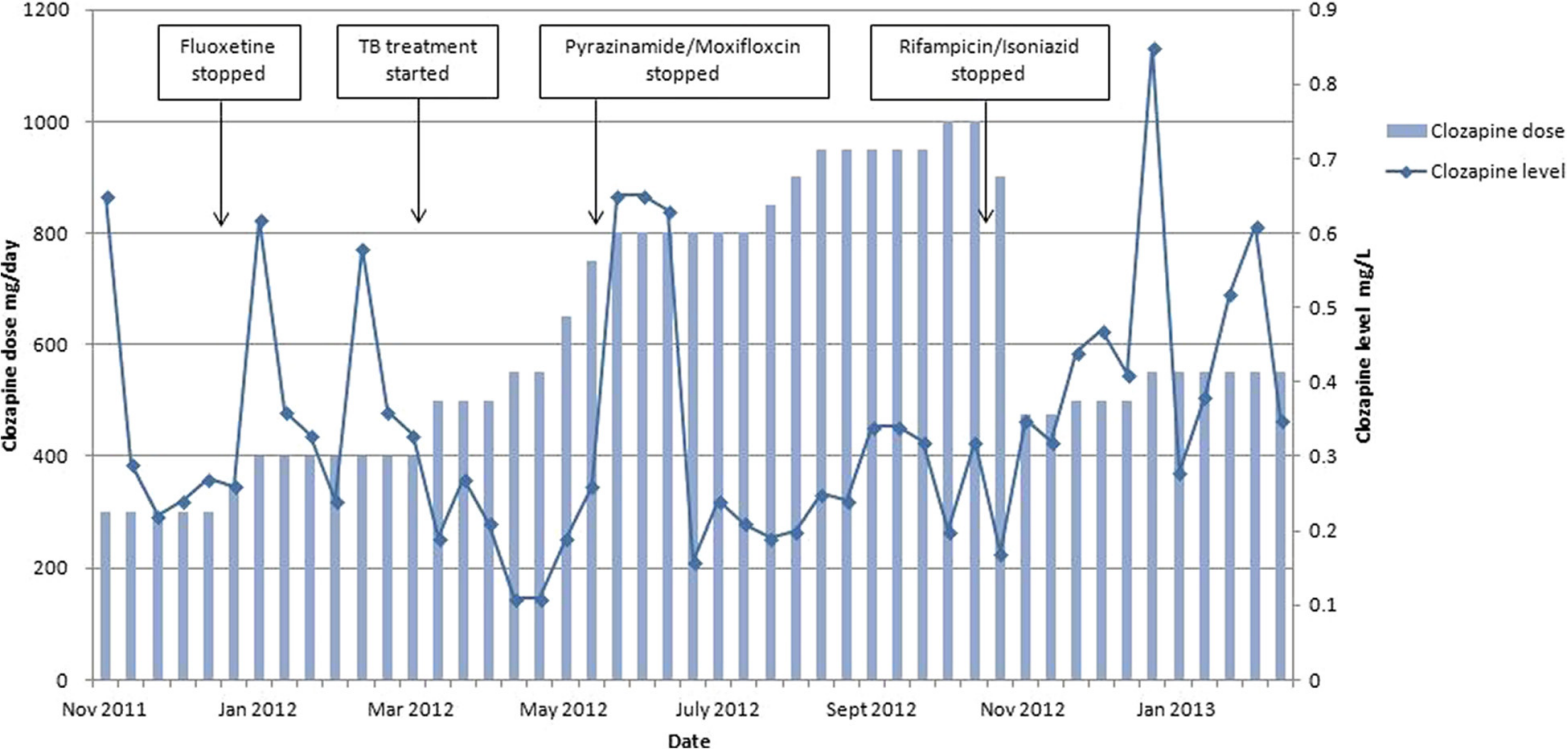
Clozapine plasma levels and the effect of medication changes

In the 5 months prior to commencing TB treatment, his clozapine levels were generally in the range 0.24 to 0.36 mg/L with the highest level being reported as 0.65 mg/L (Fig. 2). The initial clozapine dose of 300 mg/day was increased to 400 mg/day once his fluoxetine was stopped, in anticipation of a potential drop in the clozapine level following the loss of enzyme inhibition. On commencing TB treatment and in anticipation of the likely impact of enzyme induction; the clozapine dose was increased to 500 mg/day with further progressive increases over subsequent weeks. Only when the clozapine dose reached 800 mg/day after 2 months of TB treatment did clozapine levels reach the therapeutic range. This was maintained for only 3 weeks before the level again dropped. Despite increasing the clozapine dose to 1000 mg/day, clozapine levels did not reach the therapeutic range for the remainder of the TB treatment. Unfortunately this period coincided with Mr. X being very unwell with increased levels of psychotic symptoms on the catatonic spectrum including mutism, stereotypies and agitation.

On stopping TB treatment, the clozapine dose was prospectively halved by down-titrating at a rate of 50 mg/day over 10 days. The clozapine plasma level remained relatively steady and subsequent increases in dose to 500 mg/day then 550 mg/day finally led to reaching a clozapine plasma level within the therapeutic range. The degree of psychotic symptoms improved including increased spontaneous speech, reduced distraction, reduced stereotypies and increased goal directed activity.

## Discussion

This case highlights the difficulty in achieving a therapeutic level of clozapine when a powerful enzyme inducer such as rifampicin is used. Even when using a clozapine dose above the licensed maximum, the level remained sub-therapeutic. A limitation of this report is the absence of formal outcome measures but clinical observations of presentation reflected sub-therapeutic levels. Although confirmed compliance with medication is often difficult to assess in patients with psychosis, there were no concerns about compliance with medication at this time. The patient is a non-smoker, not noted to have significant intake of caffeine and no other medications known to interact with the clozapine metabolic pathways were changed during the period. His renal and liver function tests remained stable throughout the treatment period. All clozapine plasma level measurements were taken at consistent times (trough levels). It is possible that the presence of abdominal TB affected the absorption of clozapine from the gastrointestinal tract, although a comparison of absorption of isoniazid and rifampicin in patients with abdominal TB and pulmonary TB showed no differences in drug absorption for patients with abdominal TB, at least for these two drugs [41].

Given the naturalistic trajectory of drug treatment in this patient, and the clinical imperative to avoid both clozapine toxicity and sub-therapeutic levels, medication changes were made to anticipate likely effects on clozapine levels. This optimises safety but complicates interpretation of drug levels associated directly with any single medication change. On stopping the pyrazinamide and moxifloxacin component of TB treatment after 2 months there was a 3 week period of raised clozapine levels. This was the only time during the total 6 months of TB treatment that levels were above the sub-therapeutic threshold. There is no literature to suggest that pyrazinamide or moxifloxacin interact with clozapine metabolism so the cause for this remains unclear. Isoniazid is a CYP1A2 inhibitor, and is therefore expected to increase clozapine plasma concentrations [42]. It is likely that this effect was counteracted, at least in part, by rifampicin in our patient. As both drugs were initiated together, it is not possible to elucidate the extent to which isoniazid may have ameliorated the effect of rifampicin on clozapine plasma levels.

It is difficult to estimate precisely the time taken for medication changes to affect enzyme induction and de-induction and hence clozapine levels. However, 12 days after starting rifampicin treatment, the clozapine plasma level of 0.19 mg/L was lower than any preceding level since starting clozapine, despite having increased the dose by 100 mg/day in anticipation of the induction effect. This was the first level checked after starting rifampicin, and it is possible that enzyme induction was in fact quicker than 12 days and certainly faster than the 2–3 weeks reported by Joos et al. [24] Regarding de-induction after stopping rifampicin, the first available level was after 16 days and at 0.35 mg/L was at the lower limit of the therapeutic range. It was not until 4 weeks later that the level was consistently in the therapeutic range. The fact that in the interests of patient safety the clozapine dose was down-titrated immediately on stopping rifampicin makes it more difficult to interpret the effect of enzyme de-induction of clozapine.

Rifampicin is also known to affect the pharmacokinetics of lamotrigine [35]. Lamotrigine plasma levels decreased on addition of rifampicin for this patient, necessitating dose increases to maintain plasma levels within the therapeutic range. It is possible that this reduction in plasma levels contributed to the deterioration in mental state, but we consider it unlikely that this was solely responsible.

Although the proportion of TB cases reported in the UK to be resistant to at least one first line antibiotic appears to be reducing, risk factors for resistance include homelessness, substance abuse and imprisonment [22]. It is possible then that psychiatrists may be more likely to see such cases. Where resistance to the standard first-line drugs exists (isoniazid, rifampicin, pyrazinamide, ethambutol), medications such as streptomycin, amikacin, capreomycin, cycloserine, azithromycin, clarithromycin, moxifloxacin and protionamide may be used instead [1]. Considering the pharmacodynamic properties of these drugs and the available literature, interactions with clozapine are unlikely. Prescribers should be aware however that increases in clozapine concentration have been reported in combination with erythromycin, and that both clozapine and clarithromycin may prolong the QTc interval. It is also worth noting that bacterial infections have been reported to increase clozapine concentrations independently of any drug effects [43, 44] and so it may be prudent to measure clozapine plasma levels in the presence of concurrent infections, even in the absence of reported pharmacological interactions.

## Conclusions

In summary, it is appropriate to increase the dose of clozapine on commencing an enzyme inducing medication such as rifampicin. The induction effect can be seen within a matter of days and it is notable that even doubling the clozapine dose may not result in therapeutic plasma levels. . On stopping rifampicin, the clozapine dose should be reduced. As the risks of clozapine toxicity should be deemed potentially more harmful than a sub-therapeutic level, down-titration may be implemented relatively quickly. Prescribers should be aware of the significant inter-patient variability in clozapine metabolism. We suggest the following key principles be used to ensure effective and safe treatment of a psychotic illness with clozapine when co-administering a course of TB therapy:

Take clozapine plasma levels before commencing TB therapy to establish the ‘target’ plasma level for the individual patient.

Monitor clozapine levels weekly during TB therapy to determine the individual patient’s pharmacokinetic response to the drug. Alter the clozapine dose accordingly. Twice weekly plasma level monitoring in the initial weeks may be warranted.

It may be necessary to exceed the licensed maximum dose of clozapine to attain a therapeutic plasma level. It is good practice to involve a second opinion from a colleague if such a treatment plan is to be implemented. As such a strategy will be unlicensed, consent should be sought if possible from the patient, and all decisions carefully documented.

Use information from the up-titration stage to inform the speed and extent of the down-titration stage, aiming for the pre-treatment dose of clozapine at cessation of TB therapy.

Down-titration of clozapine may be carried out relatively rapidly (over 7 – 10 days), acknowledging the risks of high clozapine plasma levels. Seizure prophylaxis with an appropriate antiepileptic should be considered if plasma clozapine levels exceed 0.5 mg/L.

## Consent

The patient was formally assessed and found to lack capacity to consent to this publication. Written informed consent was obtained from the patient’s mother for publication of this case report and any accompanying images. A copy of the written consent is available for review by the Editor of this journal.
